# Is the Species Flock Concept Operational? The Antarctic Shelf Case

**DOI:** 10.1371/journal.pone.0068787

**Published:** 2013-08-02

**Authors:** Guillaume Lecointre, Nadia Améziane, Marie-Catherine Boisselier, Céline Bonillo, Frédéric Busson, Romain Causse, Anne Chenuil, Arnaud Couloux, Jean-Pierre Coutanceau, Corinne Cruaud, Cédric d'Udekem d'Acoz, Chantal De Ridder, Gael Denys, Agnès Dettaï, Guy Duhamel, Marc Eléaume, Jean-Pierre Féral, Cyril Gallut, Charlotte Havermans, Christoph Held, Lenaïg Hemery, Anne-Claire Lautrédou, Patrick Martin, Catherine Ozouf-Costaz, Benjamin Pierrat, Patrice Pruvost, Nicolas Puillandre, Sarah Samadi, Thomas Saucède, Christoph Schubart, Bruno David

**Affiliations:** 1 UMR 7138 UPMC-MNHN-CNRS-IRD « Systématique, Adaptation, Évolution », Département Systématique et Évolution, Muséum national d'Histoire naturelle, CP 39, Paris, France; 2 UMR 7208 UPMC-MNHN-CNRS-IRD « BOREA », Département Milieux et Peuplements Aquatiques, Muséum national d'Histoire naturelle, CP 26, Paris, France; 3 UMS 2700 MNHN-CNRS « Outils et Méthodes de la Systématique Intégrative », Muséum national d'Histoire naturelle, CP 26, Paris, France; 4 UMR 6540 CNRS – DIMAR « Diversité, évolution et écologie fonctionnelle marine », Université de la Méditerrannée Aix-Marseille II, Chemin de la Batterie des Lions, Marseille, France; 5 Genoscope, Centre National de Séquençage, 2, rue Gaston Crémieux, CP5706, Évry, France; 6 Royal Belgian Institute of Natural Sciences, Brussels, Belgium; 7 Laboratoire de biologie marine. Université Libre de Bruxelles, Brussels, Belgium; 8 Alfred Wegener Institute for Polar and Marine Research, Bremerhaven, Germany; 9 UMR 6282 CNRS-Université de Bourgogne « BIOGEOSCIENCES », Dijon, France; 10 Biologie 1, Institut für Zoologie, Universität Regensburg, Regensburg, Germany; Institut Pluridisciplinaire Hubert Curien, France

## Abstract

There has been a significant body of literature on species flock definition but not so much about practical means to appraise them. We here apply the five criteria of Eastman and McCune for detecting species flocks in four taxonomic components of the benthic fauna of the Antarctic shelf: teleost fishes, crinoids (feather stars), echinoids (sea urchins) and crustacean arthropods. Practical limitations led us to prioritize the three historical criteria (endemicity, monophyly, species richness) over the two ecological ones (ecological diversity and habitat dominance). We propose a new protocol which includes an iterative fine-tuning of the monophyly and endemicity criteria in order to discover unsuspected flocks. As a result nine « full » species flocks (fulfilling the five criteria) are briefly described. Eight other flocks fit the three historical criteria but need to be further investigated from the ecological point of view (here called « core flocks »). The approach also shows that some candidate taxonomic components are no species flocks at all. The present study contradicts the paradigm that marine species flocks are rare. The hypothesis according to which the Antarctic shelf acts as a species flocks generator is supported, and the approach indicates paths for further ecological studies and may serve as a starting point to investigate the processes leading to flock-like patterning of biodiversity.

## Introduction

Thanks to the International Polar Year (IPY) 2007–2009 and the Census of Antarctic Marine Life (CAML), the recent increase of oceanographic explorations across the Southern Ocean has confirmed the status of the Antarctic continental shelf as an area of rich marine biodiversity [1]–[4]. The richness of the benthic Antarctic fauna was known before [5]–[7] but considered as yet underexplored. Such a richness was observed for many benthic groups such as pycnogonids, ascidians and polychaetes [8], teleost fishes [9], echinoderms [10]–[11], crustaceans [7], poriferans and hydrozoans [12]. [8] reported that while some groups appear more diverse there than elsewhere in terms of species richness (some isopod lineages, pycnogonids, bryozoans, sponges, ascidians), others appear equally diverse (polychaetes, amphipods, echinoderms), and others less diverse (decapods, molluscs, teleosts). All these organisms are a boon for research concerning processes generating biodiversity because they have flourished within a relatively isolated area for long periods [13]–[16]. A substantial part of the marine Antarctic species richness might well be the result of species flocks, especially within the benthic fauna. Indeed, the Antarctic continental shelf has been described as a giant species flocks generator [17], as explained in more detail below. Species flocks are bursts of closely related endemic species which are ecologically diverse and numerous relatively to surrounding areas [18]. We can't observe those bursts directly, however, they are responsible for certain patterns in modern biodiversity. The first steps to study species flocks are therefore in the detection of these patterns, since knowledge of the present patterns of biodiversity is necessary prior to further exploration of the processes having generated it [16].

Eastman and McCune [17] noticed physical similarities in terms of isolation, depth and age, between the Antarctic shelf and ancient lakes where species flocks were found. Indeed currents, sub-zero temperatures and distance isolate the Antarctic shelf from all other shelves of the Southern Ocean. The Antarctic shelf is approximately 450 m deep in average [8], i.e. eight times deeper than the world shelves average, because of the weight of the ice sheet on the continent [19]. The age of the shelf geological isolation dates back at least 40 Ma, and the shelf has existed under polar conditions for 14–12 Myr [20]. Moreover, repeated advances and retreats of the ice sheet on the shelf probably caused benthic faunal extinctions [21] but also stimulated speciation events [22] through population fragmentation in isolated areas of the shelf or population displacement in refugia in sub-antarctic islands or in the deep sea [16], [23]. All these physical and historical factors are likely to have promoted species flocks at different times in a geographic area including the Southern Ocean (i.e. south of the Polar Front) and the sub-Antarctic islands. Therefore we can expect an important part of benthic biodiversity to exhibit the genetic, geographical and ecological patterns of species flocks at different taxonomic levels.

The aim of the present survey is to evaluate the hypothesis of a species flock origin among different groups of the Antarctic benthic fauna, using the criteria of Eastman and McCune [17] for species flocks detection. We mainly focused on the rich sampling of the French-Australian cruises CEAMARC (2007–2008) and the French cruises REVOLTA (2009–2012) in the Eastern Antarctic coastal waters to answer the following questions: how frequent are species flocks? How useful and suitable are the criteria used by Eastman and McCune to detect them? Consequently, is it necessary to revisite the original criteria to identify a flock? The project tries to approach these questions with four distantly related taxonomic groups of the benthic fauna: notothenioid teleost fishes, crinoids (feather stars), echinoids (sea urchins) and crustacean arthropods. Some other groups (e.g. molluscs or pycnogonids) will also be occasionally mentioned.

### What is a Species Flock?

It is important to distinguish between the theoretical definition of a species flock and the criteria to identify or detect them using empirical data. As to theoretical definitions, Ribbink [18] defined a species flock as « *an assemblage of a disproportionately high number, relative to surrounding areas, of closely related species which apparently evolved rapidly within a narrowly circumscribed area to which all the member species are endemic* ». The definition is ambiguous because it mixes empirical criteria (like high number of species, endemicity) with conjectural processes that generate species flocks (« evolved rapidly »). The former are embedded into the practice of identification, the latter are not linked to direct practice but rather belong to a theoretical scheme. Ribbink [18] did not put emphasis on monophyly while it is essential for Greenwood [24]. Starting from both definitions of Ribbink [18] and Greenwood [24], Eastman and McCune [17] focused on criteria to detect species flocks and retained five criteria: monophyly, high species diversity (called « speciosity »), high level of endemism, morphological and ecological diversity, and habitat dominance (in terms of biomass). Some of these criteria imply to determine the historical and geographic pattern of biodiversity (monophyly, endemism, speciosity), whereas others rely on ecological studies (ecological diversity, habitat dominance). The first three criteria deal with space and time, the two others with present dynamic interactions. Each of these criteria has to be assessed for a set of species in comparison to its sister lineage of the surrounding areas, especially speciosity, endemism [25], and ecological diversification. In spite of potential difficulties in gathering sufficient reliable data for each of these criteria, it is nevertheless possible to recognize sets of species that clearly correspond to species flocks (for instance the Notothenioidei at the scale of the Southern Ocean, as convincingly proposed by Eastman and McCune [17], and sets of species that clearly do not correspond to flocks (liparid fishes of the Antarctic shelf, see below). The present study will detect such conspicuous cases, but will also evaluate more intermediate situations, potentially due to difficulties in applying the criteria.

### Model Case

The standard case for the Southern Ocean has been described by Eastman and McCune [17]. Notothenioid fishes are monophyletic [26] at the scale of the Southern Ocean (including sub-Antarctic islands). Within this area they have a level of endemicity of 97%, which is exceptionally high for a marine group. The species diversity of notothenioids (134 species, according to Fishbase, [27] is very high with regard to other components of the ichthyofauna. Notothenioids represent at least 50% of it. The notothenioids are also morphologically and ecologically diversified [9], [28]. This group is benthic in origin, and secondarily diversified into niches in the water column, involving pelagic or partially pelagic zooplanktivory and piscivory [28]. The group contains benthic, epibenthic, cryopelagic and pelagic species, with morphological diversification associated to vertical motion in the water column and neutral buoyancy control rather than to diversification in trophic morphology [28]. Finally, a number of studies summarized by Eastman and McCune [17] have confirmed that notothenioids clearly dominate the fish biomass, of which they represent more than 90% [9]. The notothenioids are therefore described as a giant species flock at the scale of the whole Southern Ocean by Eastman and McCune [17], providing a model and a point of reference for our further comparisons. The question remains: can we recognize other benthic species flocks within the Southern Ocean? If some sets of species fail to meet all criteria, which are the failing criteria? How to apply the multiplicity of criteria by which we recognize a flock?

## Methods

**Figure 1:**
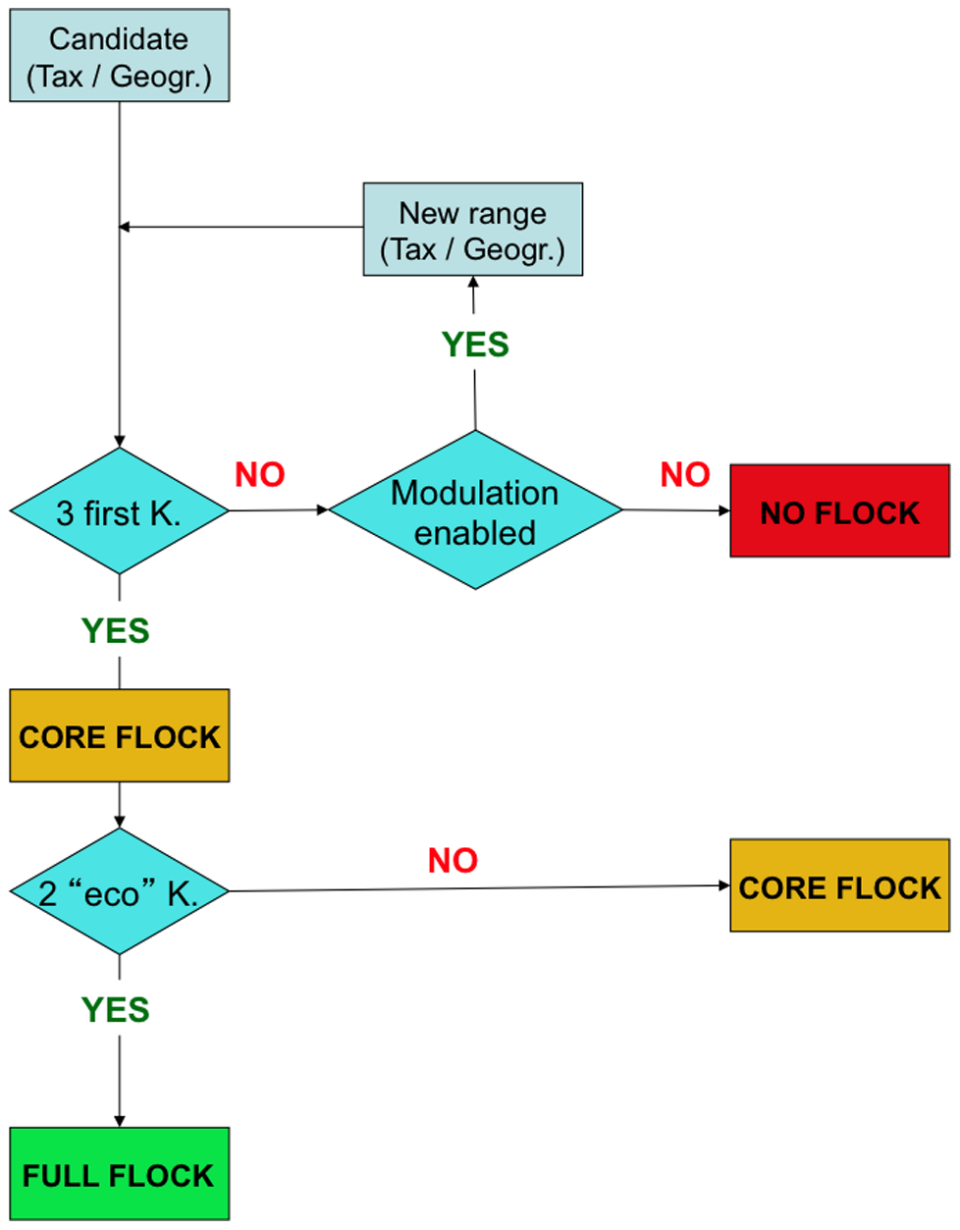
Protocol to use the five criteria of Eastman and McCune [17] to detect and evaluate species flocks. The first three criteria (3 K) are the species diversity (« speciosity ») of the taxonomic component, its level of endemicity, and its monophyly. The two other criteria (2 K) are habitat dominance (in biomass) and ecological diversity. The modulation loop means that the geographical range and the taxonomic rank may have to be redefined in order to discover unsuspected flocks.

We propose to rank the criteria in order to discover species flocks at various geographic scales within a given taxonomic group. Because evolutionary processes leading to flocks are not known or not obvious at the first glance, putative flocks are primarily recognized through the study of patterns of biodiversity: speciosity is obtained through taxonomy, endemicity from geographical distribution and monophyly from character distribution (i.e. from phylogenies, either molecular or morphological). Then, from a heuristic point of view, a flock can be primarily seen as a set of patterns (taxonomic, geographical, phylogenetic) associated with ecological diversity. The two ecological criteria are also important, but require a larger number of studies to be properly evaluated: when these criteria do not appear to be fulfilled this can be an artifact, i.e. a lack of precise knowledge hampering their assessment. The priority given here to historical criteria is therefore more practical than based on theoretical precedence. Geographic and taxonomic range seem to be the easiest criteria to assess. The question is therefore the following: at which geographic-taxonomic scales will all criteria be satisfied in their entirety? Starting from a given group in a given area as a candidate to be a species flock, we may reduce the taxonomic range, or expand the geographic one to reach monophyly, high endemism and speciosity within realistic limits, for example, those of the Southern Ocean. This corresponds to the modulation loop in Fig. 1, leading to the identification (or rejection) of a flock. A similar approach may be applied to uncover other larger or smaller flocks related to the firstly identified one. This approach should facilitate the discovery of nested species flocks. For example, the fact that notothenioids are described as a giant species flock would not prevent discovery of smaller, more recent, subflocks within the group. The two other criteria, which are more difficult to assess, can then be evaluated for the group. We provisionnally propose to call « core flock » a potential species flock for which the first three geographical and historical criteria are fulfilled, and « full flock » a potential species flock for which all the five criteria are fulfilled. Some flocks will probably remain as « core flocks » either because at least one ecological criteria failed or because there is not enough available data to assess one of these two criteria. The first three criteria are the ones that can be evaluated the most consistently. The approach allows finding where (and if) they are reached, and in which set of species. This method of assessment is not an unselective means of finding flocks everywhere. Instead, as shown below, some components of the benthic fauna of the Antarctic shelf are definitively not species flocks.

**Figure 2:**
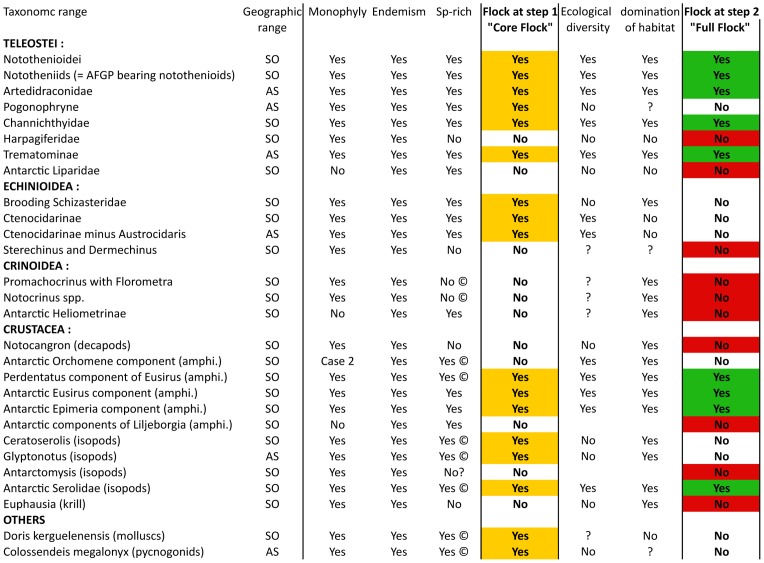
Results on species flocks estimation concerning four taxonomic components: teleosts, echinoids, crinoids, crustaceans. Amphi: amphipods, SO: Southern Ocean, AS: Antarctic Shelf, ©: newly discovered cryptic species are to be added.

Fig. 1 depicts a flow chart showing the two-step protocol used to detect species flocks. The three « historical-geographical » criteria (monophyly, endemism, speciosity) are the first ones to be assessed. It means that a failure to fulfill one of those three eliminates the taxon as a potential flock. A loop modulates the geographical range and the taxonomic rank to discover new flocks at not yet investigated levels. The two « ecological » criteria (morphological-ecological diversity, domination of habitat in terms of biomass) are considered afterwards. Results are summarized in Fig. 2.

## Results

### True Species Flocks (or « full » flocks)

The ecological and morphological diversification of the Nototheniidae, the most speciose notothenioid family (49 species) is very high and well documented [28], and the habitat domination at the scale of the Southern Ocean is not in doubt [9]. Endemicity is above 97%. However, the monophyly of the family, repeatedly considered as uncertain [29]–[32], has recently been seriously challenged [33]: four other families are embedded within it (Harpagiferidae, Artedidraconidae, Bathydraconidae and Channichthyidae). As a result, the « nototheniid flock » could actually be the flock of notothenioids with anti-freeze glycoproteins (AFGPs), i.e. notothenioids less the 13 notothenioid species of the subantarctic basal families Bovichtidae (11 species), Pseudaphritidae (1 species) and Eleginopsidae (1 species) [9], [32]. These proteins are sometimes seen as the key-innovation responsible for the sudden burst of diversification [22], [26], [34], but see [35]. In summary, there is evidence for a species flock here, but this flock should no longer be termed the « nototheniid flock », but rather the flock of the monophyletic group of notothenioids with the antifreeze glycoproteins (the « AFGP bearing notothenioids », Fig. 2, line 2). This species flock contains at least three nested flocks: the Trematominae, the Channichthyidae and the Artedidraconidae, which are detailed below.

With 13 species all endemic to the continental shelf and to some peri-insular plateaus of the Southern Ocean, the subfamily Trematominae (Teleostei, Notothenioidei, Nototheniidae) contains 10% of the notothenioids. However it is possibly underestimated because several species exhibit intra-specific chromosomal variability according to the geographic sector and sometimes within the same sector [36]–[37]. Their chromosome numbers and structures exhibit the highest diversity among the notothenioid clades. Chromosome diploid numbers and formulae differ from one species/population to another, except for *Trematomus loennbergii* which shows a highly polymorphic, unstable karyotype. They range from 2n  = 24 (*T. eulepidotus)* to 2n  = 58 (*T. nicolai*) and change according to Robertsonian fusion or fission events (reviewed in [37]). The group is monophyletic [30]–[31], [35], [38]. There is a noticeable degree of ecological diversity that does not reflect the phylogeny, i.e. niche changes do not appear to come from common ancestry but rather occur several times independently [31], [39]–[41]. The Trematoninae represent an important part of the biomass of coastal ichthyofauna [42]–[44]. Moreover they correspond to a sudden burst of diversification (to the exclusion of *Trematomus scotti* which is the sister-group of the rest of the subfamily) that occurred some 10 Ma [41], [45], though this feature is not among the criteria of Eastman and McCune [17]. Therefore the Trematominae can be considered as a smaller and more recent flock restricted to the Antarctic shelf and a few peri-insular shelves (e.g. South Georgia and South Sandwich islands [46].

The icefishes (family Channichthyidae, Notothenioidei) are endemic, monophyletic [32], [47] and display some degree of speciosity (16 species [48]) with regard to related families. They represent a very important part (more than 25%) of the Antarctic fish biomass [9]. They exhibit a noticeable ecological diversity [28], [49] linked to the ability to feed in the water column. Beyond these fulfilled criteria, it is also interesting to notice that their phyletic diversification seems to have occurred rapidly [35].

Artedidraconidae (plunderfishes) are speciose (30 species), monophyletic [29]–[30], [47] and endemic to the Southern Ocean. They show some degree of ecological diversification [50], though they need to be studied further, and possess only modest morphological diversity [9]. They are provisionally considered as a species flock [51].

The peracarid Crustacea is the most speciose animal group of the Southern Ocean, with more than 1000 strictly Antarctic species. Among them, amphipods are the most diverse, comprising 919 species in the Southern Ocean, and 547 species from the Antarctic region only (south of the Antarctic Polar Front), of which 417 species, or about 70%, are endemic [52]–[54]. In this environment, they have colonised a wide variety of ecological niches and achieved a successful eco-ethological diversification [55]. The genera *Eusirus* and *Epimeria,* though present elsewhere, each have components in the Southern Ocean that are monophyletic, given the present state of knowledge, and meet the five criteria for a species flock. A phylogenetic analysis of Antarctic *Epimeria* species confirmed a monophyletic assemblage of 26 Antarctic species and placed two potentially closely related non-Antarctic species (from New Zealand) as closest relatives [56]. *Epimeria* species are large, heavy, highly calcified and almost entirely benthic animals, with low mobility. They display a high diversity in morphology and trophic types. It is possible that the biogeographic range of *Epimeria* reflects its tolerance to a limited range of temperature, or cold stenothermy, which would provide an important clue as to its potential isolation in Antarctica from other parts of the world. To date, *Epimeria* provides the best example of a species flock within Antarctic amphipods. *Eusirus* spp. are medium- to large-sized predatory amphipods [57] with good swimming capacities [58], found between 0 and >7000 m [59] with benthic, pelagic or sympagic life styles [60]–[61]. Barnard and Karaman [59] listed 22 nominal species, of which seven have been recorded south of the Antarctic convergence. However, ongoing research indicates the existence of at least 23 described and undescribed species in the Southern Ocean [62]–[63]. The species of the group *perdentatus* which are particularly large *Eusirus* (60–100 mm), are found only south of the convergence; they include three named species and at least three multiple cryptic/pseudocryptic species [62]–[63]. The absence of dispersal across the Polar Front of the group *perdentatus* could be explained by their gigantism. It has been demonstrated that giant amphipods are restricted to waters with a maximum oxygen concentration, i.e. in truly icy waters [64]. Furthermore, all these species form a clade, which is geologically young (4–14 Ma) [63]. Ecological differentiation is present in this group, with most species benthic or benthopelagic, except for *E. propeperdentatus* which is entirely pelagic. Even though this group is only moderately diverse, it satisfies the criteria of a flock.

Although endemicity and speciosity of several Antarctic isopod lineages is quite high, few of these meet all five criteria of species flocks. One of them is the Serolidae whose Antarctic component is monophyletic and comprises about half of the known serolid species [65]. Serolid isopods exhibit a wide variety of habitat use and lifestyles. They are known from the shallow waters to the deep sea, have semi-sessile to highly mobile rafting species [66], and inhabit soft-sediment and rocky bottoms. Antarctic serolids thus also fulfill the two ecological criteria making them full species flocks whereas many subordinate clades nested within the isopods remain core flocks due to many, often pseudo-cryptic species, which are only weakly differentiated ecologically. As the rate of discovery of new species through the use of molecular methods increases, it is expected that in the future more flocks at lower taxonomic levels will be described which currently violate the speciosity requirement only due to a lack of knowledge.

### Core Flocks, but not « Full » Flocks

Some Antarctic isopod taxa appear to have radiated on the Antarctic shelf, and molecular studies have proven useful in demonstrating the presence of many morphologically similar, but genetically highly differentiated species [67]. The species inside these species complexes are monophyletic and can be locally very abundant but are generally only poorly differentiated in ecological terms, making them core but not full flocks (Fig. 2, e.g. *Ceratoserolis trilobitoides*, *C. meridionalis* and *Glyptonotus antarcticus* species complexes). Similarly species complexes are found using molecular tools in pycnogonids (*Colossendeis megalonyx* sensu lato [68]), which are not ecologically diverse. In other cases a lack of knowledge with regard to ecological diversity (like in the radiation of the gastropod *Doris kerguelenensis*, [69]) leads to the provisional conclusion of a “core flock” but not a “full flock”.

Echinoid taxonomic components fail to meet the five criteria, most of them lacking either ecological diversity or domination of habitat (Fig. 2). Two clades of echinoids fall within this category of « core flocks ». The Antarctic schizasterids consist of 27 morphologically recognized species of sea urchins. Over all a total of about 80 echinoid species have been recorded in the Southern Ocean so far, and they represent one third of the Antarctic echinoid speciosity. Molecular analyses based on several molecular markers confirm that the brooding species within this group of irregular sea urchins form a monophyletic group restricted to the Southern Ocean but not to the Antarctic shelf, some species being found in the Kerguelen Islands, Heard Island, and the southernmost part of South America. All species of the monophyletic brooding group are Subantarctic or Antarctic. Some species can locally dominate the echinoid fauna in term of abundance [70], but they display *a limited ecological diversity* (all are infaunal or partly infaunal deposit feeders) and consequently are not considered as a true flock. A recent population genetic study [71] confirms that the brooding schizasterids display highly reduced dispersal rates, a feature which may favour high speciation rates [72], independently of any key innovation.

In the regular echinoid family Cidaridae, the Ctenocidarinae form a monophyletic group of 21 morphologically recognized species that are restricted to the Southern Ocean [10], [73]. The genus *Austrocidaris* is found elsewhere in the Southern Ocean, but except for this genus, the Ctenocidarinae (without *Austrocidaris*) form a monophyletic group restricted to the Antarctic shelf. They are ecologically and morphologically well diversified. Some species are brooders while others are not, and their morphologically diverse primary spines are covered with numerous and various specific symbionts [74]–[75]. At both scales (Southern Ocean and Antarctic shelf), however, these two cidarid embedded groups of sea-urchins *fail to be significant in terms of biomass*, but this remains to be evaluated with more precision. Therefore, it is prudent to consider them as « core flocks », and not « full » species flocks.

We record as a « core flock » the *Pogonophryne* artedidraconid fish subgroup of 22 species because it is a remarkable example of a phyletic radiation without ecological or morphological diversification [9]. The morphology is so characteristic and, with the exception of the barbel, constant that the taxonomy of the group is the most difficult of all notothenioids. Their chromosome numbers and formulae are stable [76]–[78], whilst in other artedidraconids, they show some important structural interspecific changes. There has been sufficient collecting to indicate that their population densities and biomass are low: they do not exhibit habitat domination. However *Pogonophryne* are about 2-fold more diverse than any other Antarctic notothenioid genus, all having circum-Antarctic distributions and recently discovered species are coming from upper slope waters 1000–2000 m deep. So *Pogonophryne* is a large, recent, strictly benthic, non-adaptive (little morphological and ecological diversity) radiation, at a depth not colonized by most other clades of notothenioids. This is an interesting phenomenon contrasting, for example, with the adaptive radiation of trematomines on the shelf.

### Criteria for a flock not met

**Figure 3:**
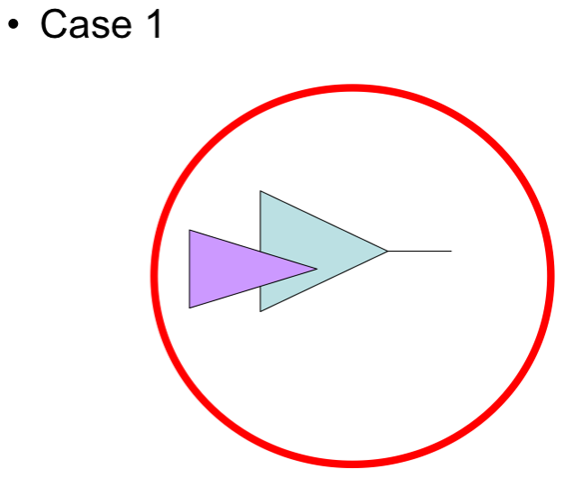
Case 1 of Table 1. A set of species is paraphyletic (blue) with a taxonomic entity embedded within (purple) it that is restricted to the area of reference (red circle): A simple taxonomic decision could fulfill the two criteria of monophyly and endemicity. Indeed the taxonomic decision would render the whole set of species monophyletic (purple becomes blue).

For crinoids, the criterion of species diversity fails and ecological diversity is often poorly documented. The two widespread and most abundant species *Promachocrinus kerguelensis* and *Florometra mawsoni* together form a monophyletic group (case 1 in Table 1, Fig. 3) distinct from other Antarctic Heliometrinae [79]–[80]. These species are not restricted to the continental shelf but are found throughout the Southern Ocean [80]–[82]. Both of them show a large morphological variability [79], [81] and live in a great diversity of habitats [79]. *Promachocrinus kerguelensis* is composed of at least seven genetic circum-Antarctic lineages [83]–[84]. However these lineages represent two seperate species at best [84]. The species diversity criterion for a flock is therefore not met. Species from the genus *Notocrinus* are dominant among brooding crinoid species [80]. This genus is monophyletic and endemic to the Southern Ocean, from the continental shelf to Burdwood Bank [80]. Molecular results indicate that the genus *Notocrinus* is composed of more than the two morphologically known species: two cryptic species within *N. mortenseni* and four cryptic species within *N. virilis* [80] are suggested. Species diversity in *Notocrinus* still needs to be precisely assessed and the present lack of knowledge prevents the assignment of this group to a species flock.

**Table 1:** Three situations for the taxon of reference (columns) are to be considered: it is monophyletic, paraphyletic or polyphyletic.

Members of the taxon present in the considered area:	Taxon:		
	Monophyletic	Paraphyletic	Polyphyletic
Monophyletic	*Flock*	*Case 1*	*No*
Paraphyletic	Case 2	Case 3	*No*
Polyphyletic	*No*	*No*	*No*

**Figure 4:**
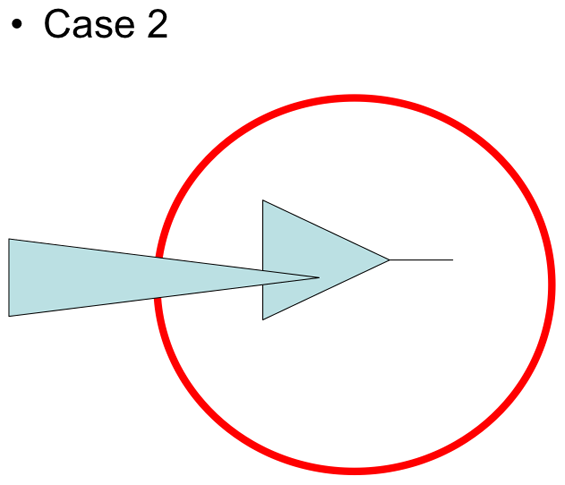
Case 2 of Table 1. The set of species under focus is monophyletic but contains an internal subpart that is secondarily « exported » outside the area of reference (red circle). See text for discussion.

There are also three situations to consider for the components of this taxon in the given area of reference (lines): monophyletic, paraphyletic or polyphyletic. To explain the table, we consider again the example of the non-notothenioid fish family Liparidae. It is monophyletic as a family (first column), however its components of the Antarctic shelf are polyphyletic because they are each related to Arctic liparids (bottom line). So the Antarctic liparid situation is the bottom left cell. Case 2 (when a taxon originating in the area of reference secondarily “exports” a part of its descent outside this area, Fig. 4) is discussed in the text.

Harpagiferids (Teleostei, Notothenioidei) are a good example of a monophyletic family not corresponding to a flock: they consist of ten species of ecologically similar fishes [9]. The non-notothenioid family Liparidae (Teleostei, Cottoidei) also does not satisfy the criteria, because of the absence of monophyly of its components inhabiting the Antarctic shelf (bottom left in Table 1). Indeed, some species of the Antarctic shelf are more related to Arctic liparids than to other liparids of the Antarctic shelf [85]–[86] (but see page 116 in [87] who considers some subgroups of Antarctic liparids as probably forming flocks).

None of the decapod or euphausiid crustaceans of the Southern Ocean studied during this survey (*Notocangron, Euphausia* respectively) are species flocks: the monophyletic components are not speciose and do not include cryptic species, though they are very important in terms of biomass (for instance the krill species *Euphausia superba* and *E. crystallorophias*). This also applies to other eucarid taxa studied to date (*Nematocarcinus* and *Chorismus* [88]).

The peracarid taxa *Liljeborgia* and *Orchomene sensu lato* are also not species flocks. *Liljeborgia* spp. are medium-sized benthic amphipod crustaceans able to swim short distances [89] and are opportunistic feeders [90], recorded between 0 and 6000 m [59]. Of the 67 nominal species of *Liljeborgia* [89], [91], 24 are known from the cold parts of the Southern Hemisphere. Of these 24, 12 are only known south of the Antarctic Convergence and three are found on both sides of the convergence [92]–[93]. A high diversity of a genus in a region can be considered as an indication of the possible existence of a local flock. However, both the examination of morphological characters and molecular data indicate that Antarctic species do not form a clade ([92], bottom left in Table 1), and suggests that they have relatives in distant seas like the Norwegian Sea [89], [93]. In other words, it is an example of a diverse Antarctic taxon, which does not fulfill the monophyly criterion. Another example of a diverse Antarctic amphipod taxon, which does not comply with the monophyly criterion, is provided by the Antarctic species of the lysianassoid genus complex *Orchomene sensu lato*. The taxon initially represented a possible candidate for a species flock in the Southern Ocean, due to its relative species diversity and high degree of endemism. This genus complex harbours at least 28 endemic, valid species in the Southern Ocean, belonging to 5 different genera. Havermans et al. [94] identified three species new to science and four species complexes each consisting of at least two cryptic species. Furthermore, d'Udekem and Havermans [95] included one more undescribed species, so that 36 potential endemic species are present in the Southern Ocean *sensu lato*, with a rate of 39% of endemicity in a total of 93 species. The Antarctic species of *Orchomene sensu lato* also exemplify habitat dominance (shelf and abyssal depths) and a significant diversity in trophic adaptations, being opportunistic or exclusive scavengers, with corresponding modifications in their mouthpart morphology [60], [96]. Furthermore, there are indications of a recent and rapid diversification of the Antarctic component of *Orchomene sensu lato* ([94]). This group of Antarctic species was initially thought to be monophyletic [96] but further studies from the same team, including more non-Antarctic species, show that this monophyletic orchomenid clade also comprised strictly Atlantic and Magellan species. However, among a majority of species restricted to the Southern Ocean, the Atlantic and Magellan taxa have a derived, more apical position in the phylogeny, which might suggest their origin within the Southern Ocean (case 2 in Table 1, Fig. 4).

In the genus *Sterechinus*, which is a regular sea urchin, a monophyletic group is obtained when the genus *Dermechinus* is included into the clade. This group is mainly restricted to the Southern Ocean, but some representatives are present both in the Antarctic shelf and in South America as well as in the Kerguelen and other subantarctic regions [97], likely due to dispersal via planktotrophic larvae. The number of species in the clade “*Sterechinus* + *Dermechinus*” (case 1 in Table 1, Fig. 3) is rather limited (weak speciosity). There are half a dozen morphological species, but molecular data (mostly mitochondrial, complemented by recent nuclear data) suggest only three to four genetic species [98], and reveal a lack of congruence between genetics and geography; monophyletic groups of species (or even of haplotypes within species) do not correspond to geographical regions.

## Discussion

### Complex cases of « Nested Flocks » and « Exportations »

In some cases, when phylogenies are available, we detect an acceleration of cladogeneses for more internal subsets of the flock. These subsets may be regarded as nested flocks. For instance, within notothenioids, AFGP-bearing species constitute a nested flock probably allowed by the key-innovation of antifreeze glycoproteins available in the blood [22], [26], [35]. In molecular phylogenies this acceleration of cladogeneses corresponds to a weak resolution (or no resolution at all) in the branching patterns among the main nototheniid lineages with regard to the crown group [31]–[32], [35], [38]. Another case is illustrated by the subfamily Trematominae that contains a diversified set of 13 species of coastal fishes, which exhibit a later acceleration of cladogeneses in the sister-group of *Trematomus scotti* [41], [45]. However, despite of the fact that most of the flocks described exhibit a period of rapid cladogeneses (soft polytomy, [99]) or a simultaneous diversification (hard polytomy, [99]), the rate of diversification is not included among the criteria of Eastman and McCune [17], while it is in Ribbink [18]. Here nested flocks show situations where *there is* an acceleration (when studied): non-*Austrocidaris* ctenocidarins within the Ctenocidarinae, Trematominae within the notothenioids [35], [41], possibly Artedidraconidae and Channichthyidae within notothenioids [35].

When using the criteria in order to detect species flocks, a diversity of situations appear which are liste in Table 1. When a set of species is monophyletic within the area of reference to which it is endemic, two criteria of species flocks are fulfilled (top left in Table 1). For instance, this is the case for the Antarctic component of the amphipod genus *Epimeria*. When a set of species is monophyletic but its members in the area of reference are polyphyletic, i.e. not closely related to each other, the monophyly criterion fails and there is no species flock (bottom left in Table 1). For instance, this is the case for liparid fishes of the Antarctic shelf: liparids are monophyletic as a family but its Antarctic members are polyphyletic because each of their diverse components is directly related to Arctic liparids. Let's consider the case 1 of Table 1 (top middle cell): when a set of species is paraphyletic (Fig. 3, blue) with a taxonomic entity embedded within (Fig. 3, purple) it that is restricted to the area of reference (Fig. 3, red circle), a simple taxonomic decision could fulfill the two criteria of monophyly and endemicity. Indeed the taxonomic decision would render the whole set of species monophyletic (purple becomes blue). This is the case for the crinoids *Promachocrinus kerguelensis*, into which the crinoids of the genus *Florometra* are embedded. Renaming *Florometra* as *Promachocrinus* would render that genus monophyletic.

**Figure 5:**
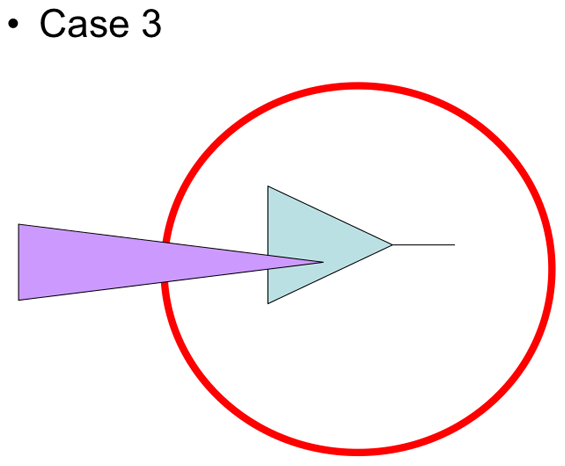
Case 3 of Table 1. This situation is a mix of case 1 and case 2. A taxonomic decision would simply lead to case 2, where supplementary data about the tempo of diversification would then be required (see text).

These situations are clearcut (italics in Table 1). Other situations are more complex. In the case 2 of Table 1 (middle left cell), the set of species under focus is monophyletic but contains an internal subpart that is secondarily « exported » outside the area of reference (Fig. 4). That pattern corresponds to the amphipod genus *Orchomene sensu lato* which Antarctic component contains an Atlantic subpart and the criterion of endemicity fails. However, viewed through time, the diversification could result from a two phases historical process: an initial burst of species and ecological diversity in a restricted area (i.e. the actual species flock), followed by an expansion of derived members of the flock outside this area. In such a case the species flock status could be maintained only when the rapid diversification is documented (for instance as in [41]). However, in the precise case of the Antarctic *Orchomene sensu lato*, the tempo of diversification remains to be investigated. Case 3 (middle cell of Table 1, Fig. 5) is a mix of case 1 and case 2. A taxonomic decision would simply lead to case 2, where supplementary data about the tempo of diversification would then be required.

Facing the case 2 of Table 1 (Fig. 4), two situations can be distinguished depending on the degree of the exportation. First, the status of species flock can be maintained by expanding the area of reference, all other criteria being satisfied, as long as the expanded new area remains within certain limits, consistent with the geographic criterion. This is an easy way, but the appraisal of a realistic expansion is a matter of empirism. In the present survey, we defined the Southern Ocean as the maximal area. For example, the icefishes (Channichthyidae) fit the definition of a species flock on the Antarctic continental shelf. Biogeography mapped onto their phylogeny (that is rather well resolved [47]–[49]) clearly shows that this benthic family originates on the shelf. However, some species are found outside the shelf (e.g. *Channichthys rhinoceratus* and *Champsocephalus gunnari* in Kerguelen Islands, *Chaenocephalus aceratus* in Bouvet Island, *Champsocephalus esox* in Magellan Strait). Consequently, the Channichthyidae is considered a species flock of the Southern Ocean.

The second way to answer is to rely on the history of the events. This is the case for deep-sea serolids of the Southern Hemisphere. Some of them occur outside the Southern Ocean (e.g. *Acutiserolis spp*. in [65]), but the molecular phylogeny places all of them firmly inside the radiation that took place in the shallow waters close to the Antarctic continent. This suggests that, subsequent to Antarctic speciations, the involved species had expanded their distribution area northward, rather than having arisen outside the Southern Ocean. This is also the case of the lysianassoid genus complex *Orchomene sensu lato* which clearly originates within the Southern Ocean, but has Atlantic and Magellan components well embedded within the Southern Ocean clade. These examples emphasize the importance of (1) reliable large-scale samplings and (2) historical information derived from phylogenetic trees. If the decision had rested exclusively on a geographic description of where the species occurred today, the monophly criterion would have been mistakenly violated even though the major evolutionary event (a major radiation occurring inside a narrowly circumscribed area) would have been true. Because a species flock is also defined by Ribbink [18] as a historical evolutionary event characterized by rapid species and ecological diversifications, the possibility for secondary exportation of some components outside the area from which it originates should be left open. This requires an early rapid diversification of the flock within the original area. Indeed the « flock » categorization within the initial geographic realm would be maintained only if we have precise information about the tempo of diversification: the « exported » subpart must originate crownward in the phylogeny of the flock, and phylogenies must exhibit early acceleration of cladogeneses at the base of the flock within the area of reference (soft polytomies being possibly due to unappropriate genetic markers or unsufficient data, the check for hard polytomies can be done using several markers separately). For example, the Antarctic species of *Orchomene sensu lato* are thought to have undergone a rapid and recent diversification [94] and the AFGP-bearing notothenioids exhibit a rapid tempo of diversification at the base of their tree [32]–[33], [35]. Among them, the genus *Patagonotothen* comprises 15 species branched crownwards, of which 14 have secondarily colonized northwards to the southern coasts of South America and the Falkland Islands [9]. Therefore, by recognizing notothenioids as a giant species flock, Eastman and McCune [17] already implicitly tolerated those secondary exportations. It is obvious that taking into account this phylogenetic-historical supplementary criterion, it may possibly contradict the initial criterion of endemicity. This discrepency is due to the fact that, even if a species flock is defined as a phyletic radiation with ecological diversification, the five criteria of Eastman and McCune [17] do not incorporate the radiation, i.e. do not estimate the tempo of phyletic diversification. This is mostly due to the fact that the species flock concept gets more complex when passing from a typological application to its historical interpretation. It is basically not a typological concept, but a historical one. The present work could lead to the recognition of a sixth criterion, fully recognizing the species flock concept as a historical one in the core of its application, the high rate of phyletic diversification, to be modulated with the criterion of endemicity, which consists in tolerating «secondary exportations » outside the area of reference.

### An amended detection of flocks

Eastman and McCune [17] noticed that the identification of species flocks in the marine realm had received less attention than in freshwater lakes or in islands. The present study fills the gap. Our practice of Antarctic and subantarctic species flocks detection in various taxonomic groups is based on multidisciplinary practical experience (joining taxonomic expertise including morphology and anatomy, field work, molecular phylogenetics and cytogenetics, field ecology) that leads us to propose an improvement of species flock recognition, aiming to make it more operational and instrumental. First, it appears necessary to focus on the three robust, easier to determine criteria: monophyly, endemism, and speciosity. We recommend ranking the ecological criteria as secondary, hence suggesting the distinction between “core” flocks and “full” flocks. No criterion is actually easy to determine: species diversity is a continuous parameter that must be compared to species diversity of the sister-group and surrounding areas and assessment of endemism and monophyly heavily depend on reliable field samplings [25]. Species flocks may be underestimated because of a lack of detection of cryptic species, but they can be overestimated through overestimation of endemism due to uncomplete field sampling. Ecological criteria are the most difficult to document, because they require even more data to be robustly appraised, and because these data are in themselves complex to obtain in the field. Last but not least, there is a component of arbitrariness in the spatial and taxonomic delineation in the estimation of « habitat dominance » or the « ecological diversity ». For instance, notothenioids represent 90% of the fish biomass of the Antarctic shelf: however it is negligible with regard to the whole eukayote biomass. « Habitat dominance » depends on the arbitrary taxonomic realm of « fishes », a non-monophyletic group. Finally, in order to have more flexibility and to assess the robustness of flocks, we suggest introducing the possibility to tune up the spatial or the taxonomic ranges in order to meet the endemism/monophyly criteria respectively. The difficulty is to maintain the adjustments within realistic limits. Those limits can be set by logical comparisons with surrounding areas as originally suggested by Ribbink [18], but so far there is no more than a rule of thumb. The confrontation of Eastman and McCune's criteria with real organisms encountered in the field compelled us to assign priorities. The flow chart of Fig. 1 should be regarded as a guide in the process leading to flock identification. It operates as a prioritized protocol, as well as a synthesis, of our practical approach to flocks. However this flow chart does not yet encompass an aspect of Ribbink's definition of a species flock: the fact that the initial phyletic diversification occurred « rapidly ». Despite not being a criterion retained by Eastman and McCune, it could be an important, albeit difficult to obtain, complementary information.
